# Establishment of an induced pluripotent cell line from Taiwan black silkie chick embryonic fibroblasts for replication-incompetent virus production

**DOI:** 10.1038/s41598-019-52282-7

**Published:** 2019-10-31

**Authors:** Jenn-Fa Liou, Wen-Ren Wu, Lih-Ren Chen, Yow-Ling Shiue

**Affiliations:** 1 Division of Physiology, Livestock Research Institute, Council of Agriculture, Tainan, Taiwan; 2 Institute of Biomedical Sciences, National Sun Yat-sen University, Kaohsiung, Taiwan; 3 Department of Biotechnology, Southern Taiwan University of Science and Technology, Tainan, Taiwan; 4 Department of Biotechnology and Bioindustry Sciences, National Cheng Kung University, Tainan, Taiwan; 5 Department of Biological Sciences, National Sun Yat-sen University, Kaohsiung, Taiwan

**Keywords:** Experimental organisms, Infectious diseases

## Abstract

The objective of this study was to establish a versatile cell line for replication-incompetent virus production and inactivation with formaldehyde to generate a model of cell-based vaccine manufacturing process. To achieve this goal, we took advantage of the easily accessed chick embryonic fibroblasts. Nine-day old chick embryonic fibroblasts were obtained and subjected to be transduced with a set of lentivirus to develop a chick induced pluripotent stem (ciPS) cell line. Morphological features, positive periodic acid-Schiff staining as well as strong immunocytofluorescence of alkaline phosphatase, intestinal (ALPI) and POU class 5 homeobox 1 (POU5F1) proteins suggested that these chick embryonic fibroblasts have been transformed into ciPS cells. Further differentiation and immunocytofluorescence assays confirmed that this ciPS cell line possesses capacities and potentials to form embryoid bodies, differentiate into all three embryonic layers: ectoderm, mesoderm and endoderm with evidence of strongly positive and specific molecular markers. Immunoblot analysis next demonstrated that through recombinant DNA technology and the 2^nd^ generation lentiviral transfer system, the goose *hemagglutinin* gene (*H5*) gene was packaged into the replication-incompetent virus and highly expressed in a bladder cancer-derived cell line, T24, after transduction. The titer of ciPS-generated replication-incompetent virus is comparable to that from the Phoenix-AMPHO cell line, which is a commercial and high productive retrovirus producer. Our study successfully established a ciPS cell line which is able to produce replication-incompetent virus, providing a new strategy for cell-based vaccine production after virus inactivation.

## Introduction

Vaccination is the most effective method of controlling viral diseases. Taking seasonal influenza for an example, there are three different influenza vaccine production technologies approved by United States of America (US) Food and Drug Administration (FDA): egg-based, cell-based and recombinant flu vaccines. A vaccine typically contains an agent that resembles a disease-causing microorganism and is often made from weakened or kill forms of the microbe, its toxins, or one of its surface proteins [Vaccines, World Health Organization (WHO), https://www.who.int/topics/vaccines/en/], such as hemagglutinin. Most seasonal flu vaccines are chicken-egg-derived in both European Union/European Economic Area and US^1^. WHO recommended developing an alternative influenza virus cultivation system and investigating mammalian cell lines^2^. Madin-Darby Canine Kidney (MDCK)^3^ and Vero (kidney epithelial cells from an African green monkey)^4^ cells were shown be specially promising cell-line candidates. In 2012, the FDA approved the first non-egg produced vaccine alternative, Flucelvax®, in the US^5^. Flucelvax® is a cell-based influenza vaccine manufacturing platform, which was developed by Novartis’s using MDCK cells to grow viruses^5^.

Cell-based have several advantages over egg-based vaccines, e.g., they (1) eliminate the need for embryonated chicken eggs from managed and biosecure flocks, (2) combine the automate upstream and downstream processes, (3) reduce the potential for contamination by viable and nonviable particulates, (4) eliminate the 4 to 6 months lead times for the organization of egg supplies, (5) have faster, high-volume start-up times for production, (6) have higher initial purity, (7) could supplement seasonal vaccine supplies when multiple strain changes are necessary, (8) would substantially increase global stockpiles of pandemic influenza vaccines^6^ and (9) have no risk of egg-allergies/anaphylaxis^7^, which is mainly caused by hypersensitivity to 4 allergens found in the egg white: ovomucoid, ovalbumin, ovotransferrin and lysozyme^8^. In fact, FDA estimated that about 20% improved efficacy for the cell-based vaccine relative to the egg-based vaccines for the 2017–2018 season^9^.

Pluripotent stem cells, i.e. embryonic stem cells^10^ or induced pluripotent stem cells (iPSC)^11^ are able to differentiate into cells of all three embryonic layers: ectoderm, mesoderm and endoderm and to form chimera or teratoma^12^. In 2006, Yamanaka’s group reported 4 crucial genes: *POU domain, class 5, transcription factor 1* (*Pou5f1*, also known as *Oct3/4*), *SRY (sex-determining region Y)-box 2* (*Sox2*), *Kuppel-like factor 4 (gut)* (*Klf4*) and *Myc, myelocytomatosis oncogene* (*Myc*), which were essential for pluripotency in mouse embryonic fibroblasts^11^. Through global gene analysis, the induced cells were confirmed to display similar transcriptional and epigenetic signature to that of embryonic stem cells^13,14^. So far, several reprogramming approaches including integrating and non-integrating methods have been applied to establish iPSCs^15^. While iPSC technology is expected to revolutionize regenerative medicine, disease modeling and drug discovery in the near future^16^, here we propose that iPSCs may serve as a tool for cell-based vaccine production (inactivated virus) with a recombinant goose influenza *H5* gene.

## Results

### Transduction of chick embryonic fibroblasts

**Figure 1:**
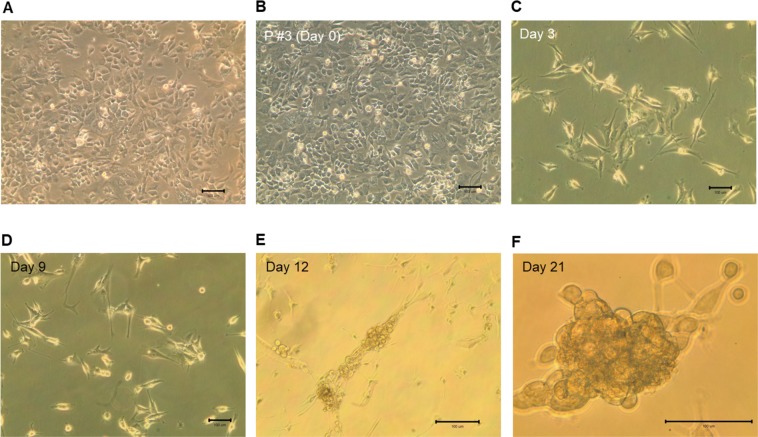
Morphologies of chick embryonic fibroblast before and after transduction with the Set of Lentivirus. (**A**) 9 days old chick embryonic fibroblasts. Chick embryonic fibroblasts (passage #3) before (**B**) and after transduction for 3 days (**C**), 9 days (**D**), 12 days (**E**), and 21 days (**F**).

**Figure 2:**
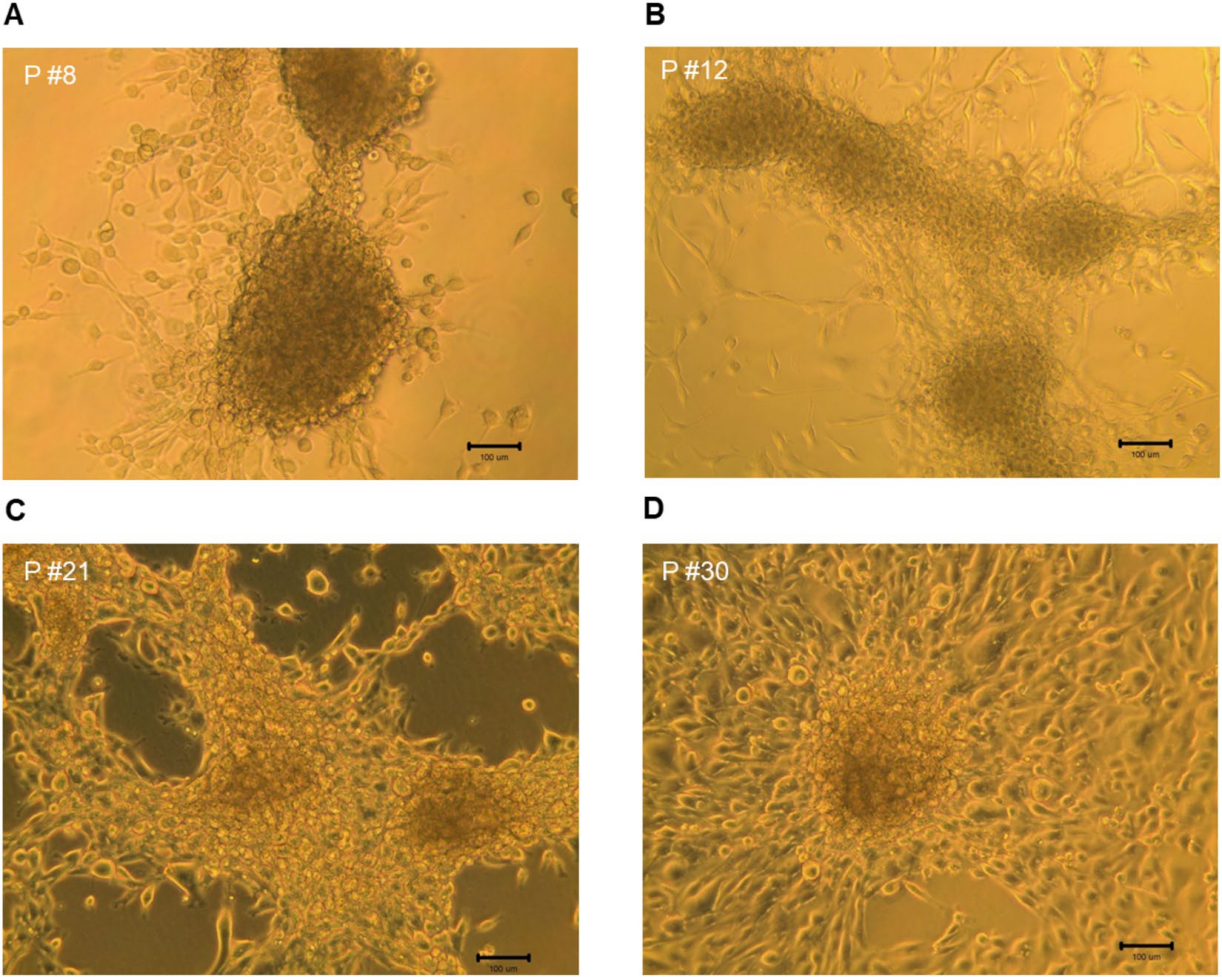
Morphologies of chick induced pluripotent stem cells at different sets of subculture passages (**A**) passage #8 (P #8), (**B**) passage #12, (**C**) passage #21 and (**D**) passage #30.

The morphology of chick embryonic fibroblasts isolated from a 9 days old chick embryo is shown in Fig. 1A. Morphologies of fibroblasts prior- (passage #3, culture for 19 days) and post-transduction for 3, 9, 12 and 21 days are also shown (Fig. 1B–F). After transduction and subculture for 5–6 days (passage #1), the shape of cells gradually turned into epithelial-like pattern (data not shown). In day 12 (passage #2), aggregation of epithelial-like cells were found (Fig. 1E). Starting day 21, embryonic stem cell-like cells were formed (Fig. 1F). In days 28 to 30 (passage #4), most cells aggregated in masses. Figure 2 shows the morphologies of transduced cells in passage #8 (P #8), #12, #21 and #30. Cells were continually subcultured to passage #35 (~300 days) and some cell aliquots were transferred to liquid nitrogen storage. Further culture of the freezing and thawing cells showed good survival and growth rates (data not shown). Although not all fibroblasts were successfully transduced by the Set of Lentivirus (*LIN28A, NANOG, SOX2, POU5F1, KLF4 and MYC*) in the beginning, only few fibroblasts were found after subculture to passage #12 to #13, due to their differentiation and unable to proliferate anymore (Fig. 2).

### Expression of chick induced pluripotent stem cells-specific markers

**Figure 3:**
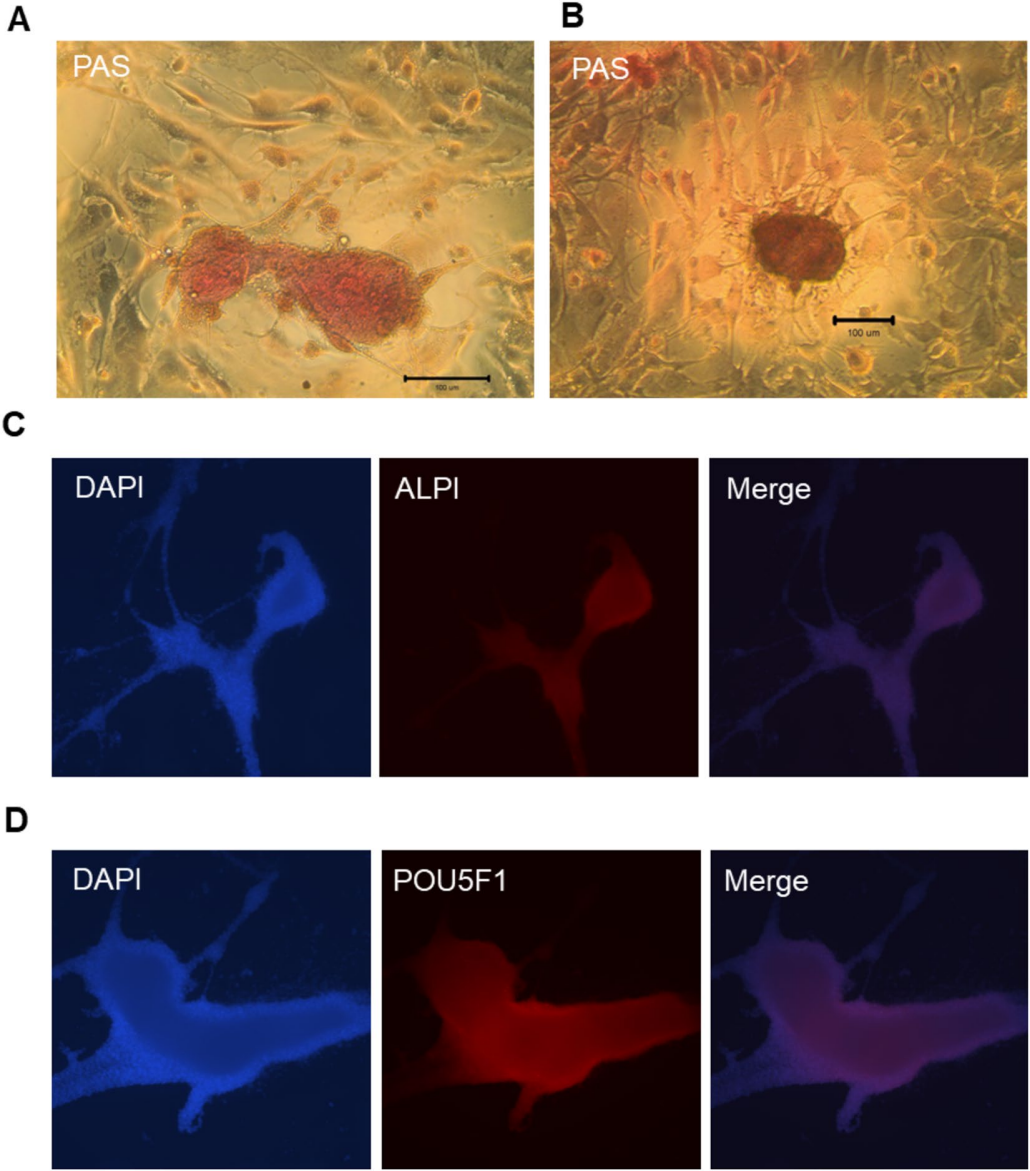
Histochemical staining and immunocytofluorescence demonstrate that chick induced pluripotent stem (ciPS) cells possess the characteristics of pluripotent stem cells. (**A**,**B**) Histochemical staining of ciPS with periodic acid-Schiff solution (PAS) after culture for 60 and 280 days, respectively. Immunocytofluorescence analysis indicated that alkaline phosphatase, intestinal (ALPI) (**C**) and POU class 5 homeobox 1 protein (POU5F1) (**D**) were highly expressed in ciPS cells after culture for 280 days. (4′,6-diamidino-2-phenylindole) (DAPI) showed the nuclear staining.

Several markers were examined to evaluate whether transduced chick embryonic fibroblasts were transformed into chick induced pluripotent stem cells (ciPS) cells. Histological staining indicated that strong positive periodic acid-Schiff (PAS) (glycogen-specific) staining in cells after culture for 60 and 280 days, respectively (Fig. 3A,B). Immunocytofluorescence further signified solid alkaline phosphatase, intestinal (ALPI) (Fig. 3C) and POU5F1 (Fig. 3D) expression levels in cells after culture for 280 days. These results suggested that a ciPS cell line has been successfully established.

### *In vitro* differentiation demonstrates the differentiation capacities of ciPS cells

**Figure 4:**
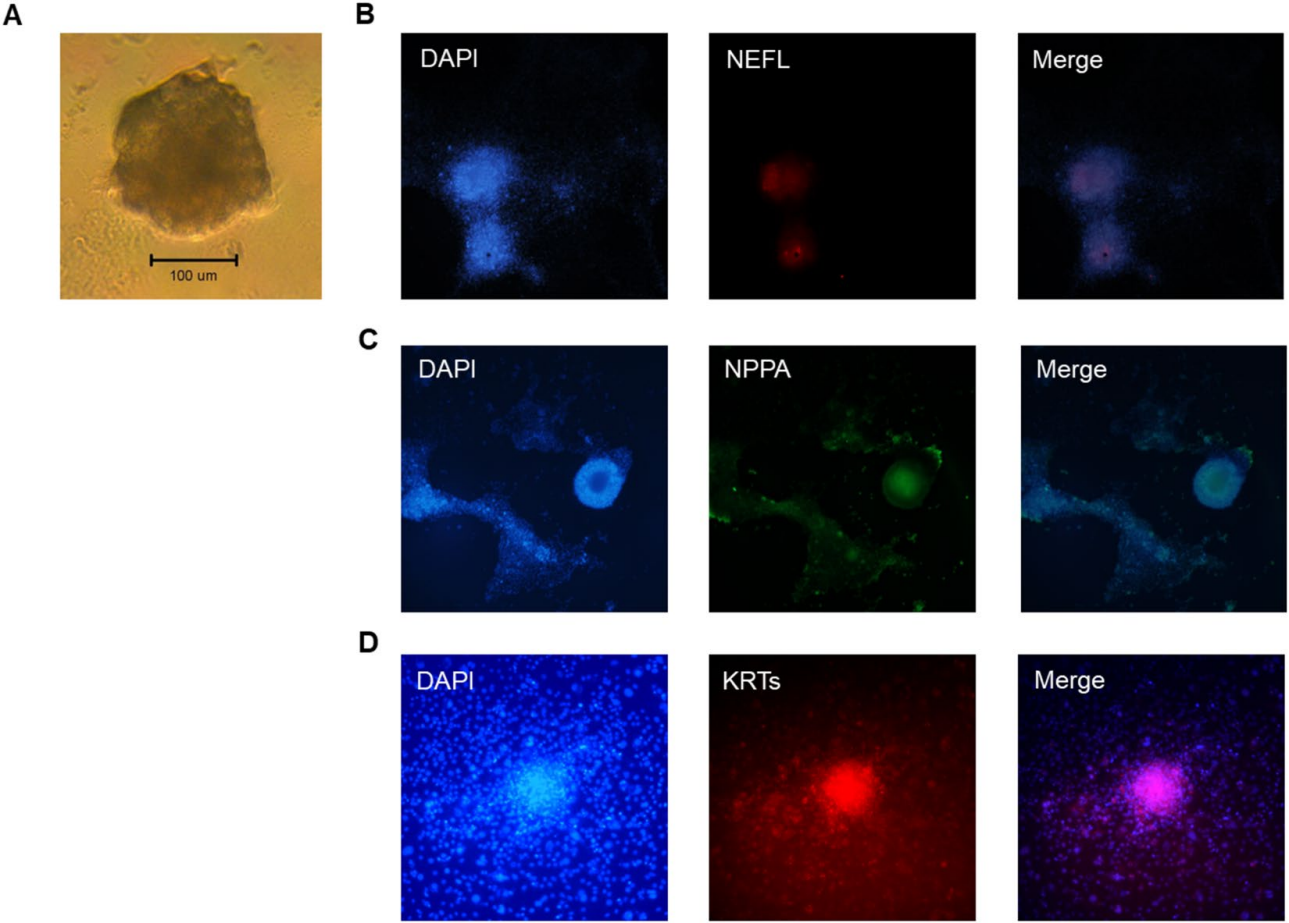
*In vitro* hanging drop/attach culture and immunocytofluorescence show the formation of embryoid body and characteristics of three embryonic germ layers in ciPS cells. Hanging drop followed by attach culture induced the formation of the embryoid body of ciPS cells (**A**). Immunocytofluorescence analysis on these embryoid bodies indicated that neurofilament light (NEFL, ectodermal marker), natriuretic peptide A (NPPA, mesodermal marker) and pan-cytokeratin (KRTs, endodermal marker) were highly expressed. (4′,6-diamidino-2-phenylindole) (DAPI) showed the nuclear staining.

As illustrated in Fig. 4A, hanging-drop culture of ciPS cells for 7 days induced the formation of ball-like embryoid bodies. The average efficiency of embryoid bodies formation was 92.6 ± 2.2% (138/150, *n* = 5) (data not shown). On day 3–5 after adherent culture, embryoid bodies attached to the surface of gelatin-coated culture plate and began to differentiate. Immunocytofluorescence with specific antibodies was performed to evaluate whether the cells were differentiated into three primary germ layers: ectoderm (marker: neurofilament light, NEFL), mesoderm (natriuretic peptide A, NPPA) and endoderm (pan-cytokeratin, KRTs). The differentiation timing of each embryonic germ layer was different. In general, neuron-like cells with Nissl bodies first appeared on day 3 after attachment. Positive staining of NEFL (Fig. 4B), NNPA (Fig. 4C) and KRTs (Fig. 4D) were identified in attached cells (Fig. 4B), suggesting that ciPS cells possess the capacities to differentiate into three primary germ layers.

### High-titer replication-incompetent virus were effectively produced in ciPS cells

**Figure 5:**
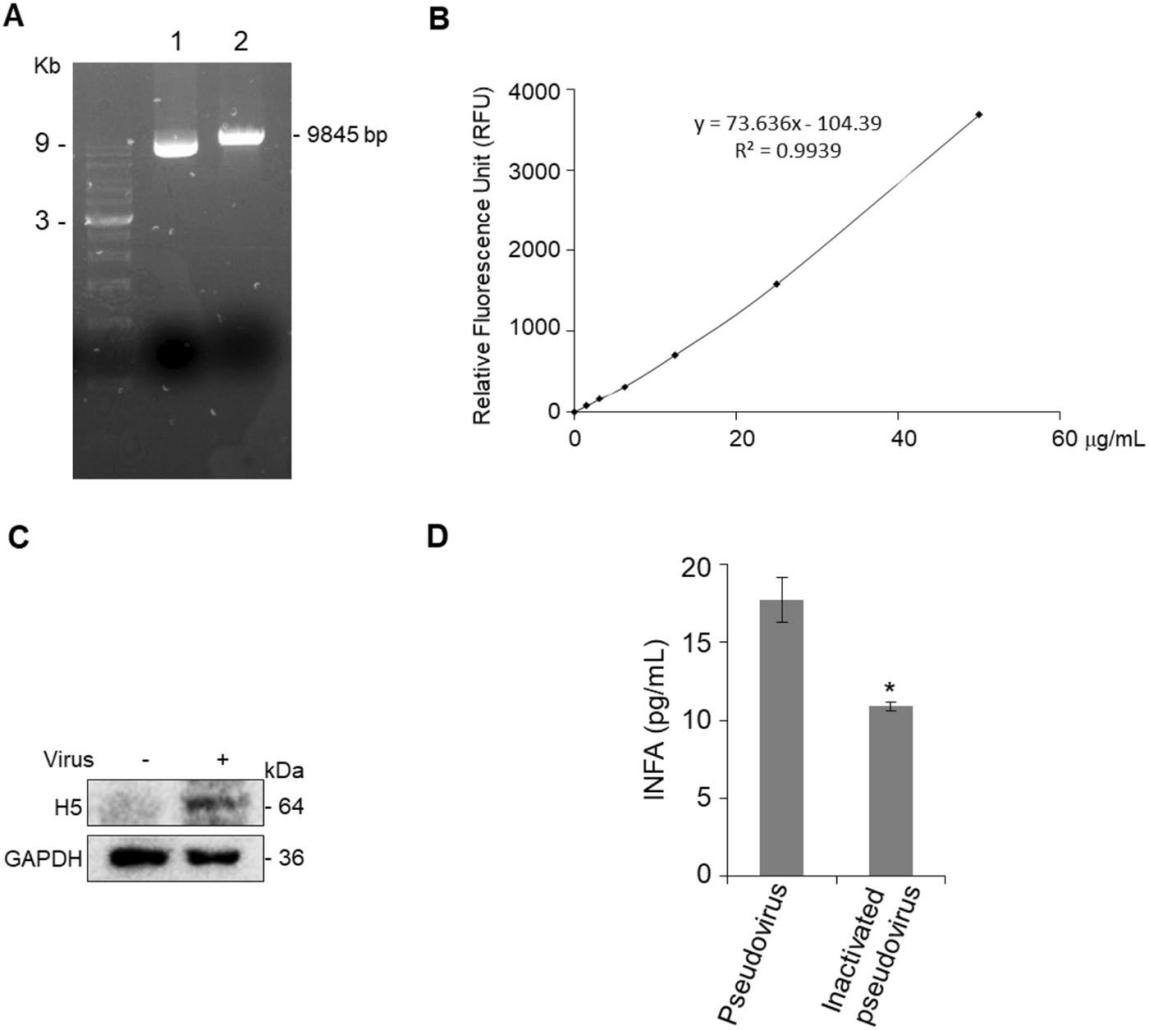
Replication-incompetent virus embracing the goose influenza *H5* gene can be produced by transduction of the ciPS cells. (**A**) The *H5* gene (1707 bp) was successfully subcloned into the pLAS2W plasmid (lane 1). Treatment with *Nhe* I enzyme linearized the plasmid (9845 bp, lane 2). (**B**) ciPS and Phoenix-AMPHO cells were seeded overnight, transfection with pLAS2W-H5, psPAX2 and PMD2G for 48 and 64 h; culture media contain replication-incompetent virus were collected. A lentivirus RNA standard curve was generated to estimate the titers of replication-incompetent virus. (**C**) One human bladder cancer-derived cell line, T24 was transduced with 1 mL of medium containing replication-incompetent virus before concentration. Cell lysates were collected and subjected to immunoblot analysis by probing anti-H5 antibody. H5 protein was notably expressed in T24 cells. (**D**) After inactivation of the replication-incompetent virus with formaldehyde at 4 °C for 24 h, the replication-incompetent virus-induced INF-alpha (INFα) was decreased compared to the non-inactivated group. All experiments were performed in triplicate and results are expressed as the mean ± SEM. For immunoblot analysis, one representative image is shown and GAPDH served as a loading control. Statistical significance: *P < 0.05.

As shown in Fig. 5A, goose influenza *H5* gene was cloned into pLAS2w.Ppuro plasmid and designated as pH5-LAS2w.Ppuro. After transfection of the pH5-LAS2w-Ppuro, *psPAX2 and PMD2.G plasmids into* Phoenix-AMPHO and ciPS cells, the media containing replication-incompetent virus were collected. The equation y = 73.636x*−* 104.39 generated using the standard curve by QuickTiter™ Lentivirus Quantitation Kit as described in the Methods, [where y is relative fluorescence unit (RFU) and x is the lentivirual RNA (ng)], was next used to estimate replication-incompetent virus particle/titer per mL (Fig. 5B). Due to the average genome size of lentivirus is 8 Kb, therefore, 1 ng lentiviral RNA = (1 × 10^−9^) g / (8000 bp × 660 g/bp) × (6 × 10^23^) = 1.1 × 10^8^ virus particle/titer. Virus titer/mL = [amount of lentiviral RNA (ng) × (1.1 × 10^8^) virus particles × (4-fold dilution)]/0.005 mL (viral volume). Before concentration, the titers of replication-incompetent virus produced from Phoenix-AMPHO and ciPS cells were estimated as 1.44 × 10^10^ and 1.34 × 10^10^ particles/mL, respectively. After concentration, the titers of replication-incompetent virus generated from Phoenix-AMPHO and ciPS cells were assessed as 2.24 × 10^11^ and 1.18 × 10^11^ particles/mL, correspondingly. Immunoblot analysis showed that *H5*-embrancing replication-incompetent virus was able to transduce T24 cells and expressed H5 protein (Fig. 5C). Treatment with formaldehyde in medium containing replication-incompetent virus and transduced T24 cells reduced interferon alpha (INFα) level in culture medium compared to non-inactivated control (P < 0.05; Fig. 5D).

## Discussion

In this study, we found that replication-incompetent virus embracing recombinant goose influenza *H5* gene could be produced in a silkie chick embryonic fibroblast-derived ciPS cell line. The titer of *H5*-containing replication-incompetent virus generated from ciPS cells was similar to which was produced from Phoenix-AMPHO cells. These results suggest that this ciPS cell line is competent for virus production. Phoenix-AMPHO is a cell line originated from HEK293 for the generation of helper-free and amphotropic retroviruses. Phoenix-AMPHO cells are highly transfectable with either calcium phosphate-mediated transfection or lipid-based transfection protocol, i.e., up to 50% or higher of cells can be transiently transfected. This cell line is capable of carrying episomes for long-term stable production of retrovirus^17,18^.

Although we used fibroblasts which were derived from fertilized eggs to establish ciPS, in the long-term culture system up to 280 days, all potential allergens in the egg white have been removed during medium changes every 2–3 days. Similar to the process of Flucelvax® production (inactivated vaccine)^19^, we inactivated *H5*-embracing replication-incompetent virus with formaldehyde. The INF level in culture medium from inactivated replication-incompetent virus-transduced T24 cells was significantly downregulated compared to non-inactivated replication-incompetent virus. Interferons (INFs) are a primary defense against pathogens due to their strong antiviral activities. There are three groups of INFs: type I, type II and type III, based on their genetic, structural and functional characteristics and receptors on cell surfaces. The type I INFs are the major group and comprise INFα, INFβ and INFε proteins, etc. The response of type I INFs to infections by immunodeficiency virus, hepatitis viruses, and influenza viruses have been well studied^20^. Significant downregulation of the INFα level in culture medium from the transduction of inactivated replication-incompetent viruses compared to activated ones, suggested that an effective inactivation was achieved, similar to the concept of an attenuated vaccine.

We provided evidence including the cellular morphology, positive staining of PAS, ALPI and POU5F, the capacity of formation of embryoid bodies, positive staining of specific markers for three embryonic germ layers in embryoid bodies as well as high replication-incompetent virus titer after transfection of the ciPS cells. All of the above suggested the potential pluripotent of this ciPS cell line and its efficiency for virus production. Among those genes [*Eukaryotic translation elongation factor 1 alpha 1(EF1A)-driven lin-28 homolog A* (*LIN28A*), *Nanog homeobox* (*NANOG*), *SOX2*, *POU5F1*, *KLF4* and bHLH transcription factor (*MYC*)] we introduced into fibroblasts to establish this ciPS cell line, *MYC* is a well-known oncogene. Indeed, immunoblot analysis showed that the MYC protein level was higher in ciPS compared to that in fibroblasts (data not shown). Using bioinformatics tools, Zhang *et al*. identified 593 iPSC consensus genes. Conspicuously, of 593 genes, 209 were also expressed in human cancer cell lines and/or tissues. Moreover, 5 oncogenes were overexpressed in the iPSCs and one oncogene *RAB25* (a member of the *RAS* oncogene family) was expressed in the iPSC-differentiated cells, signifying that these iPSC consensus genes implicated in tumorigenesis^21^. Accordingly, new strategies to downregulate the expression of oncogenic genes in iPSCs to prevent cell transformation are imperative.

To ensure the establishment of a ciPS cell line successfully, in addition to the human homologs of original mouse genes (*Oct4*, *Sox2*, *Klf4* and *Myc*) which were reported by Takahashi and Yamanaka (2006), we moreover introduced human *LIN28A* and *NANOG* genes into chick embryonic fibroblasts to establish the ciPS cell line. LIN28A is a RNA binding protein and expressed in processing bodies, where it targets mRNA and microRNA regulation. LIN28A inhibits the expression of several microRNAs including *LET-7* which regulates cell proliferation and differentiation^22,23^. LIN28A promotes the efficacy of iPSC generation in a cell cycle-dependent pathway^24^. Also, LIN28A-mediated post-transcriptional regulation of *POU5F1* expression in human embryonic stem cells^25^. Together with *NANOG*, *LIN28A* was able to replace *KLF4* and *MYC* to generate iPSCs from human normal lung cells^26^. The *NANOG* gene encodes a DNA binding homeobox transcription factor which involved in embryonic stem cells (ESCs) proliferation, renewal and pluripotency. In differentiating cells, NANOG can block ESCs differentiation^27,28^ and also autorepress its own expression in a POU5F1/SOX2-independent manner^29^. Therefore, in order to avoid the expression of oncogenes such as *MYC*, another cell line without introduction of any oncogene is under construction. Indeed, a similar approach using the same Set of Lentivirus was earlier applied to establish a chicken-induced pluripotent cell line, BA3, to support replication and growth of Newcastle disease virus LaSota vaccine strain^30^, reinforcing the potential of ciPS cells as a tool to produce cell-based vaccine. Here, we further showed that a combination of ciPS cells and recombinant DNA technology can avoid handling danger viruses in the vaccine production process.

We used the viral integrative gene delivery method to establish the ciPS cell line, which is an efficient strategy through the transgene insertions in the chromosomal DNA. However, this viral delivery system has disadvantages involving genome integration, insertional mutagenesis and lack of silencing (leaky expression)^31^. Current efforts focus on non-integrative methods such as direct delivery of synthetic mRNA of pluripotent factors (Gonzalez *et al*. 2016), transient episomal delivery along with drug selection (Jai *et al*. 2010), peptide mediated protein delivery (Gonzalez *et al*. 2016) and small molecules inducing methods^32^. In the future, iPSCs may provide an improved cell-based vaccine production, if the above mentioned methods are feasible.

In conclusion, we established a chick induced pluripotent cell line which could be used to produce high-titer replication-incompetent virus. The titer increased ~10 folds after purification and concentration. Inactivation of the replication-incompetent virus significantly downregulated the INFα level in cell culture medium after transduction, suggesting its potential for cell-based vaccine production. Specific molecular markers and the formation of embryoid body confirmed the pluripotency of this ciPS cell line.

## Methods

### Statement

All experiments and methods were performed in accordance with relevant guideline and regulations. The animal use protocol listed below has been reviewed and approved by the Institutional Animal Care and Use Committee (IACUC) at Livestock Research Institute, Council of Agriculture, Taiwan, and experiments (and relevant protocols) were performed/used in accordance with the relevant guidelines and regulations of LRIIACUC, Protocol # 106-3.

### Isolation and culture of chicken embryonic fibroblasts

All procedures were performed in sterile conditions. Fertilized eggs from a black silkie hen were incubated for 9 days and collected. Chicken embryos were washed with Dulbecco’s phosphate-buffered saline (DPBS) to eliminate the yolk and blood. The embryo was then transferred to a 10-cm Petri dish containing 10 mL DPBS to remove the head, internal organs and chopped into small pieces in a 3-mL DPBS containing 0.25% trypsin and 0.02% EDTA. In order to obtain homogenized cells, the tissue was next subjected to pass through a 18 gauge 1-inch needle with a 5-mL syringe several time and incubated in a 5% CO_2_ incubator for 5 min. Exactly 10 mL of culture medium [Dulbecco’s Modified Eagle’s Medium (DMEM, Invitrogen) containing 10% fetal bovine serum (FBS, Invitrogen, Carlsbad, CA, USA) and 1% penicillin/streptomycin (Sigma-Aldrich, St. Louis, MO, USA) was added to the tissue sample, incubated at room temperature for 5 min and subjected to be centrifuged (2000 rpm, 5 min, 5702 R, Eppendorf, Hamburg, Germany). The supernatant was removed and 10 mL medium was added, subjected to be centrifuged again. The supernatant was removed and cells were seeded at a density of 1 × 10^6^/10-cm Petri dish and incubated in an incubator with 37 °C and 5% CO_2_. Medium was renewed when cells were confluent (~70–80%) every 2 to 3 days.

### Reprogramming of the chicken embryonic fibroblasts

The Set of Lentivirus (*LIN28A*, *NANOG*, *SOX2*, *POU5F1*, *KLF4* and *MYC*] (#LV01006L, creative biogene, Shirley, NY, USA) was used to transduce cells. Each virus tube contained 10^8^ transducing units/mL. Briefly, cells were subcultured to the 2^nd^–3^rd^ passage and seeded on 3-cm Petri dishes at a density of 6 × 10^5^ overnight. Virus particles embracing the above 6 genes (3 μL/each) were mixed with 3 mL culture medium and added onto the cells. Cells were subsequently cultured in a 37 °C, 5% CO_2_ incubator for 24 h. The medium was thereafter removed and replaced with medium for chicken embryonic stem cells [DMEM with human leukemia inhibitory factor (10 units/mL, Sigma-Aldrich), human stem cell factor (5 ng/mL, Sigma-Aldrich), human basic fibroblast growth factor (10 ng/mL, Sigma-Aldrich), human insulin like growth factor 1 (10 ng/mL, Sigma-Aldrich) and human interleukin 11 (0.04 ng/mL, Sigma-Aldrich)] in the following culture and subculture. After 10 passages, mTeSR™1 medium containing 20% fetal bovine serum (FBS, STEMCELL™ TECHNOLOGIES, Taipei, Taiwan) was used to maintain the putative ciPS cell line in Corning® Matrigel® hESC-Qualified Matrix (#354277, *Corning, NY, USA*)-coated 4-well dishes. Cells were easily detached after rinse with fresh medium in the absence of trypsin and subcultured every 2–3 days.

### Characterization of the ciPS cells

To characterize whether the reprogrammed fibroblast had the features of pluripotent stem cells, PAS, ALPI and POU5F1 were detected by histochemistry and immunocytofluorescence, respectively. For PAS staining, cells were washed with PBS, fixed with 10% formaldehyde in ethanol for 5 min, washed with PBS twice, added with 1 mL periodic acid (#395B, Sigma-Aldrich), incubated for 5 min, washed with PBS for two more times, mixed with Schiff reagent (Sigma-Aldrich), incubated at room temperature for 10 min, washed with PBS twice and observed using a Axiovert 40 CFL microscope (Carl Zeiss, Oberkochen, Germany).

For immunocytofluorescence, cells were fixed with 10% formaldehyde for 30 min at room temperature, followed by incubation with 0.3% Triton X-100 for 10 min and next with 5% FBS for 2 h. Primary antibodies, anti-ALPI (1:100, #MAB4349; Millipore, Temecula, CA, USA) and anti-POU5F1 (1:100, #AB3209; Millipore) were used to hybridize the cells overnight. Rhodamine-conjugated AffiniPure rabbit anti-mouse IgG (H + L) (#315–025–003, Jackson ImmunoResearch, West Baltimore Pike, PA, USA) and rhodamine-conjugated AffiniPure goat anti-rabbit IgG (H + L) (#111-025-003, Jackson ImmunoResearch) served as secondary antibodies and observed using a DM IRB microscope (Leica, Wetzlar, Germany). 4′,6-diamidino-2-phenylindole (DAPI) was used to illustrate cell nuclei.

### Formation of embryoid body, differentiation and determination of embryonic germ layers

To form embryoid body, ciPS cells were subjected to suspending culture by hanging drops in a bacteriological Petri dish. ciPS cells were collected and cultured in 20 μL of mTeSR™1 medium containing 20% FBS on the lid of 100-mm sterile Petri dish. Cells were cultured for 7 days (5% CO_2_) and the medium was renewed every other day. At the end of culture, embryoid bodies were transferred to a 0.1% of gelatin-coated 4-well plate in the same medium for spontaneous differentiation. After culture for 14 days, formed embryoid bodies were subjected to immunocytofluorescence by probing anti-NEFL) (1:100, #AB9568, Millipore), anti-NPPA (1:100, #AB1970; Millipore) and anti-pan cytokeratin (1:100, #C2562, Sigma-Aldrich) primary antibodies, followed by hybridization with the secondary antibodies as appropriate (see also the above section).

### Plasmid, transfection and replication-incompetent virus production

Plasmids including pCMV-H5-DDK (Origene Inc. Rockville, MD, USA), pLAS2w.Ppuro (expression plasmid), pMD2.G (packaging plasmid) and psPAX2 (envelope plasmid) (Academia Sinica, Taipei, Taiwan) were obtained. pCMV-H5-DDK carrying the *hemagglutinin* gene [influenza A virus (A/goose/Guangdong/1/96) (H5N1)] (#VC102416, 1707 bp, NC_007362.1, NCBI) was subcloned into the pLAS2w.Ppuro plasmid using *Nhe* I and *EcoR* I sites to generate pH5-LAS2w.Ppuro plasmid and sequence verified. ciPS cells were subcultured (1 × 10^6^) in 6-cm dishes with 5 mL medium (mTeSR™1 containing 20% FBS) overnight. In parallel, Phoenix-AMPHO cells (1 × 10^6^) (CRL-3213™, ATCC, Manassas, VA, USA) were seeded in a 6-well plate containing DMEM medium (Hyclone, San Angelo, TX, USA) with 1% penicillin/streptomycin (Corning®) and 10% FBS overnight and subjected to be transfected as a positive control. To generate the replication-incompetent virus embracing the goose influenza *H5* gene, PolyJet™ (15 μL, #SL100688, SignaGen® Laboratories, Gaithersburg, MD, USA) was used to transfect the plasmid mixture [pH5-LAS2w.Ppuro (2.5 μg), psPAX2 (2.25 μg), PMD2.G (0.25 μg) and the medium was renewed after 16 h incubation in both cell lines.

### Purification, tittering and concentration of replication-incompetent virus particles

Media were collected at 48 h and 64 h post-transfection from both Phoenix-AMPHO and ciPS cells, mixed individually from the same cell line, filtered (0.22 μm) to remove any cell debris and aliquots of 1 mL were stored at −80 °C. The titer was measured using a QuickTiter™ Lentivirus Quantitation Kit (Cell Biolab, Inc. San Diego, CA, USA) before and after concentration. Briefly, a standard curve was generated with 200, 100, 50, 25, 12.5, 6.25, 3.125, 1.5625 and 0 μg/mL (1:2 serial dilution) of the lentivirus RNA standard in a 96-well plate followed by reading with a GLOMAX®-Multi Detection System (Promega, Fitchburg, WI, USA) using a 480/520 nm filter set. Exactly 100 μL of medium containing replication-incompetent virus particles was subjected to nucleic acid digestion, virus capture, protein denaturation, viral genome release and quantitation based on the user manual. Lenti-X concentrator (#TR30025, OriGene) was further utilized to concentrate replication-incompetent viral particles. Briefly, 1.5 mL Lentivirus Concentrator (5×) was added into 6 mL lentiviral supernatant, mixed by gentle inversion and incubated at 4 °C overnight. Thereafter, the reaction was centrifuged at 3,500 ×g for 25 min at 4 °C and the supernatant was removed carefully. The virus was resuspended in cold, sterile PBS (100 μL) by gently pipetting up and down and stored at −80 °C for further experiments.

### Transduction of the replication-incompetent virus, immunoblot analysis and inactivation

A bladder cancer-derived cell line, T24 (Bioresource Collection and Research Center, Hsinchu, Taiwan) were cultured were seeded (2.5 × 10^6^ cells) in a 6-well plate containing DMEM medium (Hyclone) with 1% penicillin/streptomycin (Corning®) and 10% FBS overnight. Cells were next transduced with 1 mL of medium containing ciPS-produced replication-incompetent virus before inactivation (1.342 × 10^10^ virus particles/mL) and selected with 4 μg/mL of puromycin for 3 days. Immunoblot analysis was performed to identify whether the H5 protein was expressed. Lastly, concentrated replication-incompetent virus (1.184 ×10^11^ virus particles) was inactivated with formaldehyde (0.02%, final concentration) at 4 °C for 24 h. QuickDetect™ INFα (human) ELISA Kit was applied to quantitate the INFα level in medium using T24 cells after transduction [5 μL medium containing non-inactivated (1.184 × 10^8^ virus particles) and inactivated replication-incompetent virus (1.184 × 10^8^ virus particles) in 95 μL culture medium] along with 8 μg/mL polybrene for 4 days.

## Data Availability

The data are shown in the main manuscript and available to readers.
